# Effect of Tramadol on Corticosteroid Receptor Function in patients with Major Depression

**DOI:** 10.1192/j.eurpsy.2022.878

**Published:** 2022-09-01

**Authors:** S. Minenko

**Affiliations:** Lvov Psychiatric Hospital, Affective Disorders, Lviv, Ukraine

**Keywords:** tramadol, diasepam, corticosteroid receptors

## Abstract

**Introduction:**

It is important to study the corticosteroid receptors hypothesis of Depression.

**Objectives:**

The goal of current study was - to test the influence of the opioid, adrenergic and benzodiazepine drugs on the immunosuppressive levels of leukocyte pyruvat dehydrogenase activity (LPDG) during the DST in patients with Major Depression and Anxiety Disorder.

**Methods:**

Patients. 40 male/ mean age, 33,1+3,2 years/ and 20 premenopausal female /35,1+1,6 years/ patients with Primary Major Depressive Episode were studied. All patients were diagnosed by a psychologist and fulfilled DSM- IV criteria for Major Depressive Episode. The DST was conducted to 60 patients with Primary Depressive Episode and 30 healthy subjects

**Results:**

In the cases of Primary Major Depressive Episode in separation with an Anxiety Disorder after TRAMADOL and DIASEPAM administration activity of LPDG increased more than 25%. Tramadol of a 50 mg dose and Diasepam of a 10 mg dose had a higher immunosuppressive effect than L-DOPA (0,5 g) on alteration of LPDG activity (more than 5 mmol/l/hour, p <0,05/.

**Conclusions:**

TRAMADOL immunosuppresssive action was higher than L-DOPA and DIASEPAM on LPDG activity in patients with an Anxiety Disorder and Major Depression. Diasepam immunosuppressive action did not correlate with positive dynamics of LPDG levels of DST in patients with Anxiety Disorder (after the 4-5 th week of Diasepam treatment). From other side, mechanism of L-DOPA action on corticosteroid receptors stimulated LPDG-activity (L-DOPA therapeutic effective dose-3 g). It means that opiate, adrenergic and benzodiazepine receptors are interactiing with each other and influencing on the corticosteroid receptors in different ways during immunosuppression.

**Disclosure:**

No significant relationships.

